# Homes Abroad for Nurses

**Published:** 1888-03-03

**Authors:** J. Wilson

**Affiliations:** 45,. Colville Gardens, W.


### HOMES ABROAD FOR NURSES.

In reply to Nurse Johnson's question about a "Home abroad for Nurses," I beg to say that one of the best managed of which I know is the Hammond Institute, Avenue Delphine, Nice. Nurses are engaged from October till May, at the rate of £36 a year, their indoor and outdoor uniform. Their travelling expenses are paid, as well as three francs a week for laundress. I know that the Home is extremely comfortable. Early application, with testimonials, should be made to the directress. I do not know of any Home that would engage for the summer months only.

45, Colville Gardens, W. J. Wilson.

